# The pCONUS2 and pCONUS2 HPC Neck Bridging Devices: Results from an International Multicenter Retrospective Study

**DOI:** 10.1007/s00062-022-01191-w

**Published:** 2022-07-11

**Authors:** L. Morales-Caba, I. Lylyk, V. Vázquez-Añón, C. Bleise, E. Scrivano, N. Perez, P. N. Lylyk, J. Lundquist, P. Bhogal, P. Lylyk

**Affiliations:** 1 Department of Neuroradiology, Hospital Universitari i Politècnic La Fe, València, Spain; 2 Department of Radiology, Hospital Universitari La Ribera, Alzira, Spain; 3 Clinica Sagrada Familia, ENERI, Buenos Aires, Argentina; 4 Department of Interventional Neuroradiology, The Royal London Hospital, Barts NHS Trust, Whitechapel Road, E1 1BB London, UK

**Keywords:** Aneurysm, Bifurcation aneurysm, PCONUS 2, PCONUS2 HPC, Wide neck

## Abstract

**Introduction:**

Bifurcation aneurysms represent an ongoing endovascular challenge with a variety of techniques and devices designed to address them. We present our multicenter series of the pCONUS2 and pCONUS2 HPC devices when treating bifurcation aneurysms.

**Methods:**

We performed a retrospective review of our prospectively maintained databases at 3 tertiary neurointerventional centers to identify all patients who underwent coil embolization with the pCONUS2 or pCONUS2 HPC device between February 2015 and August 2021. We recorded baseline demographics, aneurysm data, complications, immediate and delayed angiographic results.

**Results:**

We identified 55 patients with 56 aneurysms, median age 63 years (range 42–78 years), 67.3% female (*n* = 37). The commonest aneurysm location was the MCA bifurcation (*n* = 40, 71.4%). Average dome height was 8.9 ± 4.2 mm (range 3.2–21.5 mm), average neck width 6.4 ± 2.5 mm (range 2.6–14 mm), and average aspect ratio 1.3 ± 0.6 (range 0.5–3.3). The pCONUS2 was used in 64.3% and the pCONUS2 HPC in 35.7%. The procedural technical success rate was 98.2%. Intraoperative complications occurred in 5 cases (8.9%), 4 of which were related to the coils with partial thrombus formation on the pCONUS2 HPC seen in 1 case that was resolved with heparin. In relation to the procedure and treatment of the aneurysm the overall permanent morbidity was 1.8% (*n* = 1/55) and mortality 0%. Delayed angiographic follow-up (48 aneurysms) at median 12 months postprocedure (range 3–36 months) demonstrated adequate occlusion of 83.4% of aneurysms.

**Conclusion:**

The pCONUS2 and pCONUS2 HPC devices carry a high technical success rate, low complication and retreatment rate, and good rates of adequate occlusion. Larger prospective confirmatory studies are required.

## Introduction

Wide-necked bifurcation aneurysms (WNBA) represent a particularly challenging morphology to the interventional neuroradiologist. The use of balloon or stent-assisted coiling has classically been used to aid in the treatment of these aneurysms with Y‑stenting [1], T‑stenting [2, 3], half T‑stenting, and flow‑T stenting [4] all being used to good effect. New devices including the pCONus (phenox, Bochum, Germany) [5], the Pulserider (Pulsar Vascular, Los Gatos, CA, USA) [6], and the eCLIPs device (Evasc Medical Systems Corp. Vancouver, BC, Canada) [7, 8] have also been developed to target these types of aneurysms. More recently newer intrasaccular devices have also been developed to deal with wide necked aneurysms based on the concept of flow disruption at the neck [9, 10] as well as an advancement on the traditional intrasaccular coil [11].

Some of these devices represent an advancement of the previously described waffle-cone technique [12] and they all share the common feature of providing extra coverage at the aneurysm neck to prevent coil prolapse into the parent vessel and with respect to the pCONUS and PulseRider devices there is no need to catheterize the daughter branches unlike with the eCLIPs device or stenting/balloon remodelling techniques.

The pCONUS2 is a second generation device neck-bridging device with six petals on the crown and radio-opaque markers on each of the petals to allow accurate positioning within an aneurysm. The design of the pCONUS2 allows the crown to articulate and hence accommodate steep angles between the parent vessel and the aneurysm. The pCONUS2 HPC device has a hydrophilic surface coating with reduced thrombogenicity. To date there are only a handful of publications documenting the use of the pCONUS2 and pCONUS2 HPC devices [13–16].

In this study we present our multicenter experience with the pCONUS2 and pCONUS2 HPC devices and present the technical success rate, complication rate, clinical and angiographic follow-up results. This is the largest publication to date on the use of the pCONUS2 and pCONUS2 HPC devices.

## Material and Methods

We retrospectively reviewed our database of prospectively collected data to identify all patients who presented to the interventional neuroradiology department in three centers and underwent assisted coil embolization with either the pCONUS2 or the pCONUS2 HPC device. Between February 2015 and August 2021 we identified all patients treated with the pCONUS2 or pCONUS2 HPC devices.

All patients gave informed consent and the use of the pCONUS2 or pCONUS2 HPC device was at the discretion of the operator. All patients were treated with the patient under general anesthesia. Prior to treatment the patients received dual antiplatelet therapy with aspirin and clopidogrel. Platelet function was tested in all patients using the Multiplate device (Verum Diagnostica, Munich, Germany) and if there was poor platelet inhibition to clopidogrel the patient was switched to ticagrelor following which they were retested to ensure response to ticagrelor. Standard doses for the medications were used: 75 mg per day for aspirin to be continued for life with either 75 mg per day clopidogrel (if responsive) or 90 mg twice daily ticagrelor, continued for 6 months postprocedurally.

A standard 8 Fr right common femoral approach was used for all cases. Patients were fully heparinized with a 5000 IU bolus dose of heparin and repeated bolus doses of heparin to maintain the ACT at 2–2.5 times normal. Rotational angiography was used to accurately ascertain the anatomy of the aneurysm, afferent artery and any vessels derived from the neck of the aneurysm. This was also used to correctly size the device to both the afferent artery and the aneurysm neck/base. In all cases the pCONUS2 or pCONUS2 HPC was delivered first and then the device crossed with the coiling catheter. The pCONUS2 or pCONUS2 HPC was not detached until satisfactory coiling of the aneurysm had been achieved.

### Clinical Events

Any clinical event that occurred in the postoperative period was recorded. A neurological assessment was performed prior to the treatment, after the treatment, at discharge and at follow-up.

### Radiological Assessment

Standard Towne’s and lateral angiographic images were acquired for all patients prior to treatment in addition to rotational angiographic in order to determine the most suitable projection to allow visualization of the afferent artery, the aneurysm and any branches that needed preserving. Control angiography was performed at the end of the procedure.

Aneurysm measurements were made using standard methods. The maximum fundus width, neck width and height measurements were recorded using standard techniques.

**Fig. 1:**
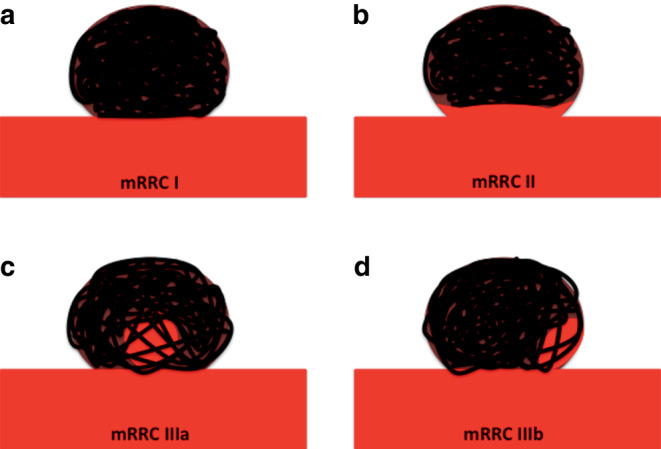
Diagram to illustrate the modified Raymond-Roy classification. Complete occlusion and neck remnants represent mRRC I and II, respectively (**a**, **b**). Grade IIIa (**c**) occlusion represents filling between the coil interstices centrally within the aneurysm, whereas grade IIIb (**d**) represents filling between the interstices and the wall of the aneurysm. Grades I and II mRRC were taken as adequate occlusion

Immediate aneurysm occlusion at the end of the procedure was based upon the modified Raymond-Roy classification (mRRC). The mRRC scale is a 4-point scale that records the occlusion of aneurysms as complete (mRRC 1), neck remnant (mRRC 2), contrast filling seen between the coil loop interstices (mRRC IIIa) and contrast filling seen between the coil loop interstices and the aneurysm wall (mRRC IIIb) (Fig. 1; [17]). The mRRC I and mRRC II were considered to be adequate occlusion.

Postprocedural imaging was performed according to the protocols of each hospital. In one hospital initial catheter angiography was performed at 3–6 months with a further catheter angiography performed at 12–18 months followed by yearly MRA. In the other hospitals catheter angiography was performed at 12 months with MRA performed yearly thereafter.

## Results

### Baseline Characteristics

We identified 55 patients with 56 aneurysms (17 patients from HULA [Hospital Universitari La Ribera] and HUPLF [Hospital Universitari i Politècnic La Fe] and 38 patients were added from CSF [Clinica La Sagrada Familia, ENERI]) with median age 63 years (range 42–78 years) and the majority of whom were female (*n* = 37, 67.3%). The majority of aneurysms were found incidentally when imaging was performed for non-specific symptoms such as headache (*n* = 43, 78.2%), stroke, TIA, seizures accounting for 9.1% (*n* = 5), and SAH, either acute or sub-acute (treated < 6 weeks from ictus) accounting for 12.7% (*n* = 7).

### Aneurysm Characteristics

The vast majority of the aneurysms were located at the MCA bifurcation (*n* = 40, 71.4%) with those arising from the anterior communicating artery (AComA) accounting for the second most common location (*n* = 10, 17.9%). The average dome height was 8.9 ± 4.2 mm (range 3.2–21.5 mm) and the average neck width was 6.4 ± 2.5 mm (range 2.6–14 mm). The average aspect ratio was 1.3 ± 0.6 (range 0.5–3.3). The vast majority of aneurysms had not been previously treated (*n* = 48, 85.7%). The pCONUS2 was used for the treatment of 36 aneurysms (64.3%) and the pCONUS2 HPC was used to treat the remainder (*n* = 20, 35.7%). The results are summarized in Table 1.

**Table 1:** Summary of demographic and aneurysm characteristics

Characteristic	Number (%)
**Demographics**	*n* = 55
**Female**	37 (67.3)
**Age**	63 years (range 42–78 years)
**Presentation**	
*Headache/asymptomatic*	43 (78.2)
*Stroke/TIA/seizures*	5 (9.1)
*SAH*	7 (12.7)
**Aneurysm Features**	
*Dome height*	8.9 ± 4.2 mm (range 3.2–21.5 mm)
*Neck width*	6.4 ± 2.5 mm (range 2.6–14 mm)
*Aspect ratio*	1.3 ± 0.6 (range 0.5–3.3)
**Location**	
*L*	22 (39.3)
*R*	23 (41.1)
*Midline*	11 (19.6)
MCA Bif	40 (71.4)
Acom	10 (17.8)
Pcom	1 (1.8)
ICA Bif	2 (3.6)
BA Bif	1 (1.8)
Pericallosal	1 (1.8)
Ophthalmic	1 (1.8)

## Technical Success and Immediate Angiographic Results

The pCONUS2 could be deployed in all cases; however, in one aneurysm the device could not be positioned correctly and therefore, this aneurysm was treated with X‑stenting (stents traversing from right A1 to left A2 and vice versa) and coils instead, resulting in an overall technical success rate of 98.2%.

**Fig. 2:**
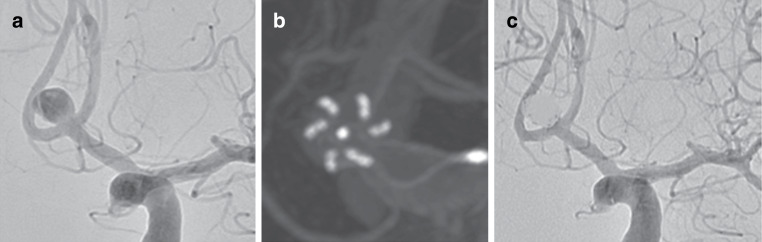
A patient with an unruptured aneurysm of the AcomA artery (**a**). A vaso CT reconstruction performed intraoperatively demonstrates the position of the pCONUS2 (4 × 15 × 6 mm) providing complete coverage at the neck of the aneurysm (**b**). Complete occlusion (mRRC 1) seen at the end of the procedure (**c**)

On angiography at the end of the procedure complete occlusion of the aneurysm was seen in 51.8% of aneurysms with neck remnant seen in 19.6% resulting in an adequate occlusion of 71.4% at the end of the procedure (Fig. 2).

## Delayed Angiographic Results

**Fig. 3:**
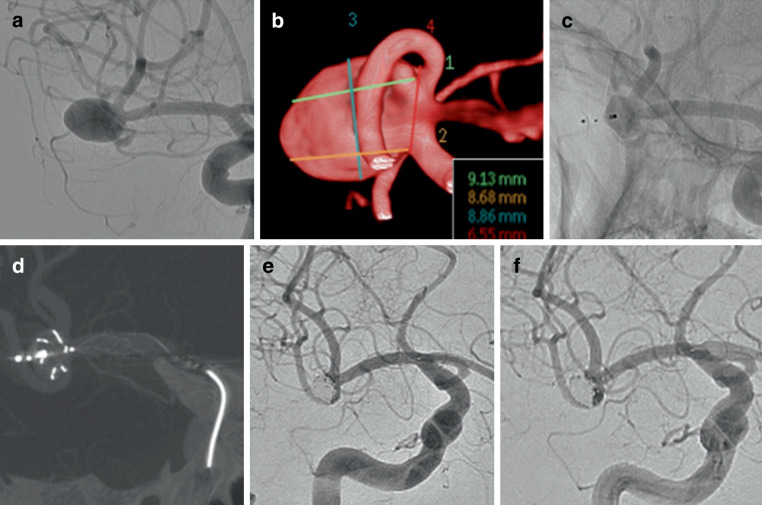
A patient with a wide necked aneurysm arising from the right MCA bifurcation (**a**,**b**) that was initially treated with a WEB device. Delayed angiography revealed a significant neck recurrence due to compression of the WEB (**c**) was treated with a pCONUS2 (**d**) and coils with complete occlusion of the aneurysm remnant on angiography performed at the end of the procedure (**e**). Delayed angiography performed at 6 months revealed stable occlusion (**f**)

Last angiographic follow-up, either MRA (*n* = 14) or DSA (*n* = 34), was available for 48 aneurysms at median time of 12 months postprocedure (range 3–36 months). At follow-up 68.8% of aneurysms were completely occluded and 14.6% had neck remnants resulting in adequate occlusion seen in 83.4% of aneurysms (Fig. 3). The follow-up results are summarized in Table 2.

**Table 2:** Summary of immediate and delayed clinical and angiographic results

Characteristic	Number (%)
**Angiographic occlusion**	
**Immediate**	***n*** **=** **56**
I	29 (51.8)
II	11 (19.6)
IIIa	8 (14.3)
IIIb	7 (12.5)
NA	1 (1.8)
**Delayed**	***n*** **=** **48**
*DSA*	34 (70.1)
*MRA*	14 (26.9)
I	33 (68.8)
II	7 (14.6)
IIIa	3 (6.2)
IIIb	5 (10.4)
**Retreatment**	***n*** **=** **55**
Yes	4 (7.3)
No	49 (89.1)
Pending	2 (3.6)

## Retreatment

**Fig. 4:**
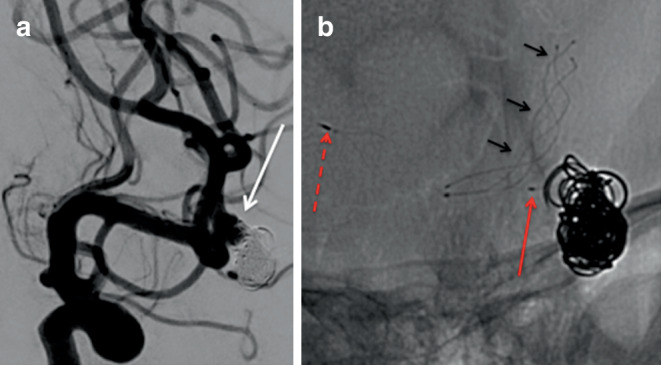
A patient previously treated with a pCONUS2 for an MCA bifurcation aneurysm was seen to have a growing lobule arising from the superior aspect of the aneurysm (**a**, *white arrow*). An LVIS Jr. (Microvention) (**b**, *short black arrows*) was placed through the pCONUS2 and across the neck of the aneurysm and lobule with additional coiling. The proximal marker of the pCONUS2 can be seen in the M1 segment of the MCA (**b**, *dashed red arrow*) and the proximal marker on the crown of the pCONUS2 can also be seen (**b**, *solid red arrow*)

In total 4 patients required retreatment of the aneurysm and in all cases the aneurysm was recorded as either mRRC IIIa or IIIb. In three of these cases this was done with additional coiling and in one case stent-assisted coiling was performed (Fig. 4).

Two patients are awaiting retreatment. There were no cases of rupture following treatment. The overall rate of aneurysms requiring retreatment (completed and pending) was 10.7%.

## Complications

### Intraoperative Complications

We observed intraoperative complications in 5 cases (8.9%). In two cases, during implantation of the framing coil, the aneurysm ruptured. This was managed with further coiling and there were no new clinical symptoms in either patient. In a further two cases the coils could not be detached properly and required fixation with carotid stents. We observed partial thrombus formation on the pCONUS2 HPC in a single case and this resolved completely with an additional bolus of IV heparin. In one case the pCONUS2 could not be accurately positioned and therefore, X‑Stent and coiling was performed.

### Periprocedural Complications

A change in the baseline NIHSS score (< 4 points) was observed in 3 patients. There was no evidence of intraoperative thrombus formation in any of these cases and in all cases the symptoms had resolved completely by the time of discharge and MR imaging performed after the symptom onset revealed small cortical DWI lesions within the vascular territory of the treated vessels. All patients remained asymptomatic at follow-up (mRS 0).

In another patient, 2 days after discharge following treatment of an MCA bifurcation aneurysm with a pCONUS 2 device, the patient presented with severe left hemiparesis and slurred speech. A left MCA thrombosis was disclosed on CT angiography. The patient was given IV rtPA by the stroke team on call, as time window, ASPECTS score and CT perfusion imaging were favorable despite the patient being on DAPT and having had recent endovascular surgery. After the bolus dose there was an immediate hemorrhagic transformation. The patient remained severely disabled, although able to walk with assistance and speak at the 90-day follow-up examination (mRS 4). The cause of the stent thrombosis was uncertain; however, the patient was receiving ticagrelor and given the time scale of the thrombosis it is feasible that an interruption to this medication was the cause. The patient was responsive to the ticagrelor on the preoperative antiplatelet testing but this was not rechecked when the patient was admitted.

In relation to the procedure and treatment of the aneurysm the overall permanent morbidity was 1.8% (*n* = 1/55) and mortality 0%.

## Discussion

We report the largest series to date on the use of the pCONUS2 or pCONUS2 HPC devices for the treatment of intracranial aneurysms. We demonstrate a good safety profile with permanent morbidity of 1.8% and 0% mortality. Technical success of the procedure was high (98.2%) with a low retreatment rate, both pending and completed, of 10.7%.

To date there are only a few publications dealing with the pCONUS2 or pCONUS2 HPC devices, two of which are case reports [13–16, 18]. The initial publication reported the immediate and early angiographic results from a single center. The device was implanted in 12 patients with aneurysms located in both the anterior (*n* = 10) and posterior circulations (*n* = 2). The majority of aneurysms were unruptured (*n* = 10). The average aneurysm dome width was 8.83 ± 5.3 mm (range 3.8–20 mm) with neck width being 5.88 ± 2.92 mm (range 2.77–11 mm). A single patient had transient neurological symptoms postoperatively; however, this was self-limiting and the patient returned to baseline neurology (mRS 0). There were no cases of mortality related to use of the device. The immediate posttreatment angiography showed complete occlusion (mRRC I) in 10 aneurysms and neck remnant (mRRC II) in the 2 remaining aneurysms. Early delayed angiographic follow-up at 4.6 months (range 3–6 months) was available in 6 patients and of these 4 showed stable and complete occlusion (mRRC I), a small neck remnant in 1 patient remained stable (mRRC II) and in 1 patient there was deterioration in the radiological appearance with recanalization of the aneurysm (mRRC IIIb).

More recently a larger series, including the pCONUS2 HPC device was published. The pCONUS2 HPC device has a hydrophilic polymer coating that has shown reduced thrombogenicity when applied to nitinol [18–25] and cobalt-chromium surfaces [26]. Yeomans et al. [16] reported the results of 20 aneurysms, 4 of which were ruptured, treated with the pCONUS2 (*n* = 13) or the pCONUS2 HPC (*n* = 7) with all of the ruptured aneurysms treated using the pCONUS2 HPC. The average age of the patients was 59 yrs old with 75% of patients being female. The average aneurysm width and height were 10.1 and 8.4 mm, respectively with average aspect ratio of 1.2. Of the aneurysms 12 (60%) were located in the anterior circulation with the remainder arising from the basilar tip. The majority of the unruptured aneurysms (13/16, 81.3%) were treated with the pCONUS2 with the remaining unruptured aneurysms treated using the pCONUS2 HPC. All of the ruptured aneurysms were treated using the pCONUS2 HPC. The authors reported neither periprocedural neurological morbidity nor mortality. A single groin hematoma with retroperitoneal extension that required surgical management was reported. These results are similar to our own with 0% mortality and only a single patient with permanent morbidity (1.8%). At 6 months, 16 patients had undergone follow-up MR angiography and this showed satisfactory occlusion (mRRC I or II) in 94% of cases (*n* = 15/16). All patients had stable neurology and mRS at this time point. Delayed imaging (24 months) was available for 6 patients with satisfactory occlusion seen in 83.3% (*n* = 5/6). In our own series delayed imaging revealed adequate occlusion in 83.4%.

Although there are no previous publications documenting use of the pCONUS2 HPC, the early experience with the pCONUS HPC has been published. Aguilar Perez et al. [22] documented their initial use of the device in conjunction with single antiplatelet therapy (SAPT) using aspirin during the treatment of ruptured aneurysms. They identified 15 patients (60% female) with median age 54 years (range 27–81 years). Intraprocedural thromboembolic complications were noted in 3 patients but none of the patients had clinical or radiological sequelae and all were managed intraoperatively. Another patient had transient aphasia postprocedure secondary to multiple small lesions seen on DWI MRI; however, at 2 days postoperatively they were asymptomatic. Angiography performed at the end of the procedure showed 66.6% of aneurysms were adequately occluded. On follow-up imaging (5 months), available for 11 patients, adequate occlusion was seen in 45.5% with no evidence of stent thrombosis or in-stent stenosis. There were no device-related delayed thromboembolic complications. Subsequently the same group published a further case series on their experience of treating unruptured aneurysms using the pCONUS HPC with SAPT in a staged manner [27] with implantation of the pCONUS HPC first and coiling performed at a later date. In total 15 patients were treated (66% female) with average age of 69 years (range 41–76 years). All patients underwent platelet inhibition testing preoperatively. The authors’ reasoning for this staged approach was that partial endothelialization of the device would increase the stability of the pCONUS HPC and hence potentially enable a more dense coil mesh to be formed. In total there were 5 complications, 4 of which were thromboemboli arising from the coils and managed with eptifibatide and one ICA dissection caused by the guide catheter. At the end of the coiling procedure adequate occlusion was seen in 60%, which increased to 80% on the early follow-up (median 168 days postoperatively). In our own series all patients received DAPT and although a staged approach was not taken in our series it does represent an alternative treatment strategy. Similarly, in two recent papers on the use of the SAPT in conjunction with the p48 HPC flow diverter the authors found a 20% incidence of asymptomatic DWI lesions in patients treated with prasugrel [28]. In comparison SAPT with aspirin resulted in 42.8% of patients having ischemic symptoms [29]. The exact reason for the difference is unknown; however, it is known that SAH and other pathologies cause platelet activation that occurs principally via the GPIIb/IIIa pathway [30] and therefore, GPIIb/IIIa and P2Y12 antagonists may be better suited for use in patients with aneurysmal SAH and in general when using HPC-coated devices.

It would not be unreasonable to assume that the placement of a stent directed towards the aneurysm may actually direct blood flow into the aneurysm. Using model aneurysms Aguilar-Perez et al. performed a series of experiments using DSA optical flow imaging techniques to assess the changes in intra-aneurysmal flow after the placement of a Solitaire AB stent (Medtronic), in a waffle-cone manner, and a pCONUS device. After the placement of either device there was actually a small decrease in the intra-aneurysmal flow. Although this study assessed the original pCONUS and not the pCONUS 2 device the results are likely to be similar given that the pCONUS2 provides increased metal coverage at the neck of the aneurysm and even less metal within the parent artery.

The PulseRider (Cerenovus) is an alternate neck-bridging device. It is fully retrievable and comes in either T or Y configurations and in a variety of different sizes. Initial case reports and small case series suggested a good safety profile and occlusion rate [31–34]. Subsequently, the results of the adjunctive neurovascular support of wide-necked aneurysm embolization and reconstruction trial (ANSWER) were published [35]. This was a prospective, non-randomized, single-arm multicenter, core laboratory adjudicated US study (*n* = 34). Mortality attributable to the device or procedure was 0%, 8.8% complication rate and a 6-month mRS ≤ 2 was achieved in 32 patients (94.1%). Satisfactory aneurysm occlusion was seen in 87.9% of cases at 180-day follow-up. These results are similar to our own with adequate occlusion in 83.4% seen on delayed imaging but with a lower rate of complications at 1.8% and no mortality attributable to the aneurysm treatment. There is, as of yet, no equivalent surface modified or coated device and therefore, DAPT remains the default position when using the PulseRider.

The eCLIPs bifurcation remodeling system is another novel neck bridging device. There is limited clinical data available for the device [7, 8]. The device has a “spine-rib” design with 2 types of ribs emanating from the spine, with some of the ribs being more densely arranged and acting as a flow disruptor across the neck of the aneurysms and the more low-density ribs acting as an anchor in the daughter branch. In the most recent multicenter publication on this novel device (*n* = 24) the authors reported a high rate of technical success (94%) with high rates of immediate adequate occlusion (80%), which increased on delayed follow-up (95%, *n* = 21).

Our study has several limitations inherent with a retrospective study as well as the fact that the sample size is relatively small and that the results were self-adjudicated. Delayed follow-up beyond 2 years is missing and therefore, the stability of aneurysm occlusion is difficult to assess. In addition, the paucity of data regarding ruptured aneurysms means it is difficult to extrapolate the safety of this device to the acute situation.

## Conclusion

The pCONUS 2 device represents an alternative to existing neck bridging devices with improved neck coverage and the ability to cope with sharply angled aneurysms. Larger prospective studies with long-term follow-up clinical and radiographic data are required to determine the safety of the device and the stability of aneurysm occlusion.

## Abbreviations

AcomA: Anterior communicating artery
ICA: Internal carotid artery
MCA: Middle cerebral artery
MED: Medina embolic device
MRRC: Modified Raymond Roy classification
MRS: Modified Rankin score
PCA: Posterior cerebral artery
PcomA: Posterior communicating artery
RRC: Raymond Roy classification
WEB: Woven endobridge
